# Increase in cases of malaria in Mozambique, 2014: epidemic or new endemic pattern?

**DOI:** 10.1590/S1518-8787.2016050006105

**Published:** 2016-03-01

**Authors:** Jorge Alexandre Harrison Arroz

**Affiliations:** World Vision Mozambique. Malaria Project Global Funded. Maputo, Moçambique

**Keywords:** Malaria, epidemiology, Endemic Diseases, Spatial Analysis, Epidemiological Surveillance, Cross-Sectional Studies

## Abstract

**OBJECTIVE:**

To describe the increase in cases of malaria in Mozambique.

**METHODS:**

Cross-sectional study conducted in 2014, in Mozambique with national weekly epidemiological bulletin data. I analyzed the number of recorded cases in the 2009-2013 period, which led to the creation of an endemic channel using the quartile and C-Sum methods. Monthly incidence rates were calculated for the first half of 2014, making it possible to determine the pattern of endemicity. Months in which the incidence rates exceeded the third quartile or line C-sum were declared as epidemic months.

**RESULTS:**

The provinces of Nampula, Zambezia, Sofala, and Inhambane accounted for 52.7% of all cases in the first half of 2014. Also during this period, the provinces of Nampula, Sofala and Tete were responsible for 54.9% of the deaths from malaria. The incidence rates of malaria in children, and in all ages, have showed patterns in the epidemic zone. For all ages, the incidence rate has peaked in April (2,573 cases/100,000 inhabitants).

**CONCLUSIONS:**

The results suggest the occurrence of an epidemic pattern of malaria in the first half of 2014 in Mozambique. It is strategic to have a more accurate surveillance at all levels (central, provincial and district) to target prevention and control interventions in a timely manner.

## INTRODUCTION

**Figure 1:**
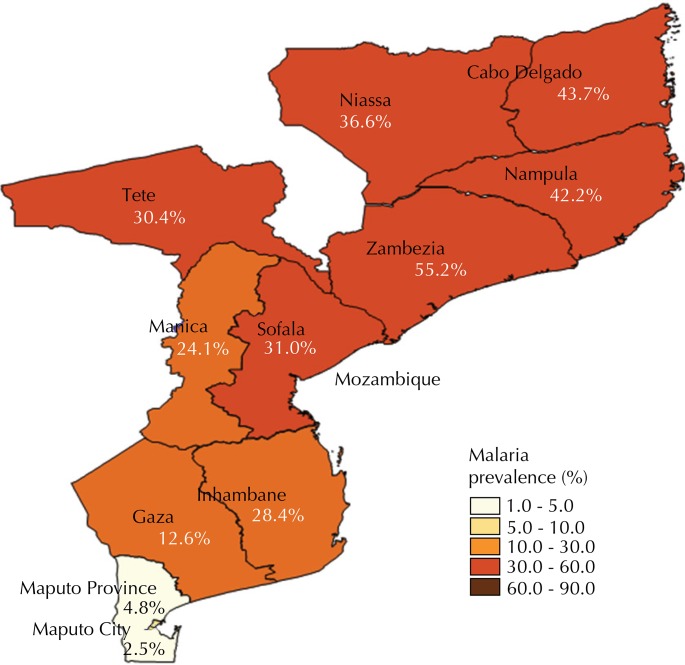
Prevalence of malaria per provinces. Mozambique, 2011.

Malaria is endemic in Mozambique, representing 45.0% of all cases observed in the outpatient consultation and approximately 56.0% of admissions in pediatric wards^a^. According to the latest demographic health survey carried out in 2011 (IDS 2011), the prevalence of malaria in children aged six to 59 months is of 35.1%, with the provinces of Zambezia and Nampula being the ones with the highest prevalence (55.2% and 42.2%) and Maputo City and Maputo Province with the lowest prevalence (2.5% and 4.8%) (Figure 1)^b^.

The main vectors of malaria in Mozambique belong to groups *Anopheles funestus* and *An. Gambiae*. *Plasmodium falciparum* is the most frequent parasite, responsible for approximately 90.0% of all malaria infections, while infections by *Plasmodium malariae* and *Plasmodium ovale* are observed in 9.0% and 1.0%, respectively^a^.

Several factors contribute to this endemicity, from the climatic and environmental conditions – such as temperatures and favorable rain patterns – to suitable locations for the reproduction of the vector, socio-economic situation of poverty-related populations, inappropriate housing and limited access to means of prevention^a^.

Despite the encouraging coverage of key control interventions, in particular, 2,252,192 mosquito bed nets distributed in campaigns, 89.0% of bed net covers for pregnant women, and 79.0% of indoor residual spray coverage (IRS), the report of the National Malaria Control Program (NMCP) for the first semester of 2014 showed that there were 3,297,386 case notified and 1,937 deaths in the entire country, an increase of 41.0% in relation to the cases reported in the same period in 2013. The same report mentions a slight reduction of the malaria-related deaths (2.0%) for the same period of analysis^c^.

The objective of this study was to describe the increase in cases of malaria in Mozambique.

## METHODS

Mozambique is situated in the Southern area and on the East coast of Africa. It has an area of approximately 799,380 km^2^, with borders to the North with Tanzania, to the West with Malawi, Zambia, Zimbabwe, and South Africa, to the South with Swaziland and South Africa, and to the East with the Indian Ocean^1^. The country comprises a wide coastal strip with an approximate extension of 2,515 km, lapped by the Indian Ocean. It presents hot and wet season from October to March (characterized by rainfall, high temperatures and high relative humidity) and a cool, dry season from April to September. However, the climatic conditions vary according to the altitude. The daily values of relative humidity varies between 10.0% and 90.0%. Average temperatures vary from 20°C in the South and 26°C in the North, and the amounts are higher during the rainy season^a^

The country has a population of around 26,564,648 inhabitants (on September, 2014)^d^ and is divided into 10 provinces and one province capital. The provinces in the North region of the country are: Niassa, Cabo Delgado, and Nampula; the provinces of the Central region are: Zambezia, Tete, Manica, and Sofala; the Southern provinces are: Inhambane, Gaza, Maputo province and Maputo city (Figure 1).

A descriptive cross-sectional study was conducted in September 2014, with secondary data from the Weekly Epidemiological Bulletin (WEB). WEB is a tool from the health information system for epidemiological surveillance in Mozambique, focused on the major infectious diseases that constitute a public health problem in the country. It is used in all health units in the country, and the produced data is aggregated at the district, province, and national level for analysis and to establish decision-making processes and actions in a timely manner.

Using the WEB, during the 2009-2013 period, the data was collected for months and the endemic channel to the period was built. This channel is a graph that allows establishing the pattern or trend of a disease over time, and thus detect patterns of elimination or control of an epidemic of the disease in question. In this study, we adopted two methodologies for the construction of the endemic malaria channel for the 2009-2013 period: quartiles method and cumulative sum method (C-Sum)^e^

I used the quartiles method to set the epidemic threshold. Data on cases of malaria have been converted into rates, using the population as denominator according to the year in question and expressed for 100,000 inhabitants. It has been estimated the 1st, 2nd and 3rd quartile, with the following zones: zone of success (below quartile 1); safety zone (between quartile 1 and 2); danger/alert zone (between quartile 2 and 3); and epidemic zone (above quartile 3).

The C-Sum method was used for the control method of the quartiles. For the calculation of the threshold for a given month, we added the previous month and the following one to the month in question, and the result was divided by 15. For example, to calculate the threshold in February, we added the data from January, February and March, and divided the result by 15. The result is a graph with a continuous line (C-Sum line) that determines the epidemic threshold. Values above the line C-Sum are considered epidemic.

Incidence rates recorded monthly were calculated for the first half of 2014, allowing to determine the pattern of occurrence of malaria during this period. The months in which the rate was higher than the third quartile by the method of the quartiles and above the C-Sum line were considered epidemic months.

Using the NMCP data related to the first half of 2014, I calculated the incidence and cumulative mortality recorded per province, which allowed to determine the provinces that were most affected by malaria.

## RESULTS

In the first semester of 2014, the provinces of Nampula, Zambezia, Sofala, and Inhambane accounted for 52.7% of all cases of malaria in Mozambique and the provinces of Nampula, Sofala, and Tete, responsible for 54.9% of the malaria deaths. The fatality rate ranged between 0.02% (Niassa) to 0.11% (Nampula and Maputo City) (Table).

**Table:** Cases, deaths and fatality rate for malaria, according to provinces. Mozambique, 1st half of 2014.

Province	Cases		Deaths		Fatality rate
		
	n*	%	n	%	%
Nampula	565,542	17.2	603	31.1	0.11
Zambezia	443,311	13.4	247	12.8	0.06
Sofala	365,121	11.1	213	11.0	0.06
Inhambane	364,498	11.1	180	9.3	0.05
Manica	363,340	11.0	173	8.9	0.05
Gaza	291,922	8.9	173	8.9	0.06
Cabo Delgado	253,351	7.7	123	6.4	0.05
Tete	243,332	7.4	80	4.1	0.03
Niassa	241,162	7.3	57	2.9	0.02
Maputo Province	131,936	4.0	52	2.7	0.04
Maputo City	33,871	1.0	36	1.9	0.11

Mozambique	3,297,386	100	1,937	100	0.06

Source: NMCP (National Malaria Control Program) – report for the 1^st^ half of 2014.

* Cases of infection by *Plasmodium falciparum.*

**Figure 2:**
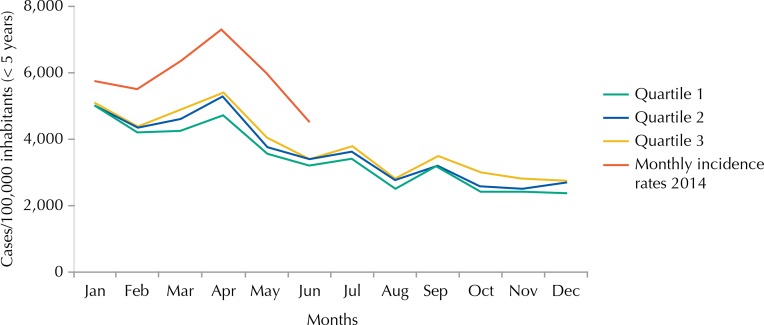
Endemic channel 2009-2013 (children under 5 years of age) by the quartile method and monthly incidence rates recorded in 2014 (children under 5 years of age), Mozambique.

**Figure 3:**
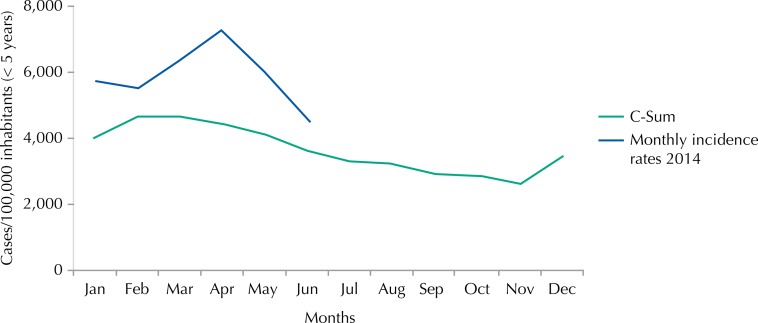
Endemic channel 2009-2013 (children under 5 years of age) by the C-Sum method and monthly incidence rates recorded in 2014 (children under 5 years of age), Mozambique.

The incidence rate of malaria registered in children under five years old shows values in the epidemic zone, rising from 5,739 cases per 100,000 inhabitants in January 2014 to 4,508 cases per 100,000 inhabitants in June of the same year, with a peak of incidence rate of 7,275 cases per 100,000 inhabitants in April (Figures 2 and 3).

**Figure 4:**
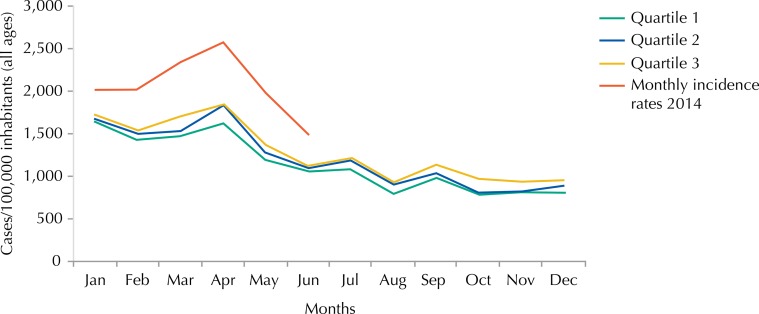
Endemic channel 2009-2013 (all ages) by the quartile method and monthly incidence rates recorded in 2014 (all ages), Mozambique.

**Figure 5:**
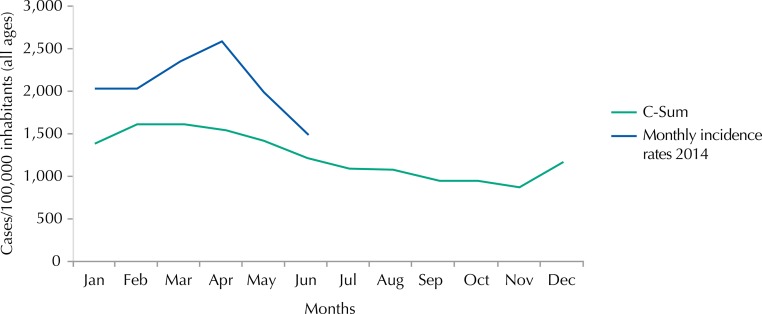
Endemic channel 2009-2013 (all ages) by the C-Sum method and monthly incidence rates recorded in 2014 (all ages), Mozambique.

The incidence rate of malaria registered for all ages shows values in the epidemic zone, rising from 2,020 cases per 100,000 inhabitants in January 2014 to 1,482 cases per 100,000 inhabitants in June 2014, with a peak of incidence rate of 2,573 cases per 100,000 inhabitants in Abril (Figures 4 and 5).

## DISCUSSION

The provinces of Nampula and Zambezia are the most affected by malaria, which can be related to the coastal provinces with favorable socio-economic and climatic factors (the most populous provinces). The findings of this study are consistent with national data, the 2011 IDS, which reveals the highest national prevalence for those provinces (Zambezia, 55.2% and Nampula, 42.2%)^b^. Several studies support a greater transmission dynamics of malaria in coastal regions of Africa^1^ ^,^ ^2^ ^,^ ^8^ ^,^ ^9^. According to the results, there was an epidemic of malaria pattern since January 2014, with a peak in April. The malaria epidemic pattern is the result of major changes in the eco-epidemiological system, with excessive rains, end of prevention measures against malaria, or loss of effectiveness of the measures adopted for any reason (resistance of mosquitoes to insecticides, gaps in implementation, financial breaks to support malaria-related control activities)^6^ ^,^ ^8^. Mozambique has little data available for an accurate analysis of the influence of climatic factors. However, the chance of a larger preview of the portion of the iceberg above the clinical horizon cannot be ruled out.

Since 2006, NMCP has reinforced the system of malaria notification through the improvement of the registration and notification of cases confirmed by laboratory or rapid diagnostic test (TDRm)^f^. In 2013, NMCP made 10,547,052 TDRm available to test about 22 million inhabitants. Now, in 2014, the amount was higher (17,374,342), which may have led to increased testing capacity, and thus the positive results are recorded and reported (larger preview of the upper portion of the iceberg)^f^. According to the score card for the elimination of malaria in Africa from the ALMA (African Leaders Malaria Alliance)^g^, Mozambique should have notified seven million cases in 2012 (which corresponds to at least three and a half million cases in a semester), showing that even this increase still represents an underreporting of the real cases. In an analysis of the epidemic curve, we can observe that it displays a pattern similar to the endemic channel, which supports the maintenance of the same seasonal trend, but in epidemic range. This suggests the increased ability to diagnosis and register due to the greater availability of diagnostic tests. The increase of the health network does not constitute a good reason, as it expanded by only 1.0% in the first half of 2014 compared with the same period in 2013, and corresponds to an increase of 344 weekly epidemiological bulletins received (Annual Report of NMCP 2014)^e^.

The various purposes of an epidemiological surveillance system include: detect epidemics; document the spread of diseases; prevent and control the occurrence of adverse events to health; and recommend the necessary measures to prevent or control the occurrence of specific harms to health with objective and scientific bases.

The mere collection and sending data is not seen as the purpose of surveillance^4^. NMCP in Mozambique has a weekly epidemiological surveillance system integrated with WEB, covering all districts and health facilities. However, the data is not analyzed systematically in the district and provincial levels. Another limitation is that these data do not reflect the actual incidence of malaria in the country, being only the registered incidence, which assumes that many cases still go undiagnosed and unregistered in health information systems.

Even an endemic channel for malaria is not available at all levels of attention, which leads to late detection of epidemics or the risk of not detecting. Several countries adopt monitoring systems combined with routine data from hospitals and climate data (precipitation, relative humidity, temperature) for the successful prediction of the rise of cases^3^ ^,^ ^5^ ^,^ ^7^.

The results suggest the occurrence of an epidemic pattern of malaria during the first half of 2014 in Mozambique. The Northern and Central Mozambique (Nampula, Zambezia, and Sofala) were the most affected areas, and that contributed significantly to the increase in incidence rates noted. The provinces of Inhambane and Gaza are the main contributors to the increase in the incidence rate registered in the Southern region of the country.

The results also suggest the strategic importance of a surveillance that is more accurate and at all levels to target prevention and control interventions in a timely manner. The districts and provinces must track their endemic channels and continually monitor the reporting of cases of malaria to make a more intense surveillance of malaria. This monitoring would extend to central level to provide greater support in promotional and preventive measures, the areas of epidemic risk patterns, and transform the malaria surveillance in a main intervention associated with universal access in the prevention, diagnosis, and treatment of cases. Basic research to boost innovation and the development of new and improved tools for epidemiological surveillance are necessary and should be placed as a main topic of a national research agenda on malaria.

## Footnotes

a Ministério da Saúde (MZ), Direcção Nacional de Saúde Pública. Programa Nacional de Controlo da Malária: Plano Estratégico da Malária 2012-2016. Maputo; 2012 [cited 2015 Sept 10]. Available from: http://www.rollbackmalaria.org/files/files/countries/mozambique2012-2016.pdf

b Instituto Nacional de Estatística (MZ); Ministério da Saúde (MZ). Inquérito demográfico e de saúde 2011. Maputo; 2013 [cited 2015 Sept 10]. Available from: http://dhsprogram.com/pubs/pdf/FR266/FR266.pdf

c Ministério da Saúde (MZ), Direcção Nacional de Saúde Pública. Programa Nacional de Controlo da Malária: relatório do 1º semestre de 2014. Maputo; 2014.

d Countrymeters. Contagem da População de Moçambique [cited 2014 Sept 15]. Available from: http://countrymeters.info/pt/Mozambique

e WHO. Epidemiological Approach for Malaria Control: Guide for tutors. 2nd.; 2013 [cited 2014 Sept 15]. Available from: http://apps.who.int/iris/bitstream/10665/96351/1/9789241506014_tutors_guide_eng.pdf.

f Ministério da Saúde (MZ), Direcção Nacional de Saúde Pública. Programa Nacional de Controlo da Malária: relatório anual de 2014. Maputo; 2014.

g Aliança dos Líderes Africanos contra a Malária – ALMA. Cartão de Pontuação ALMA 2030 para a Eliminação da Malária [poster].Genebra: WHO; 2014 [cited 2015 Sept 10]. Available from: http://www.alma2015.org/sites/default/files/sadc-elimination-scorecard/alma_scorecards_poster_portuguese

## References

[1] Bejon P, Williams TN, Nyundo C, Hay SI, Benz D, Gething PW (2014). A micro-epidemiological analysis of febrile malaria in Coastal Kenya showing hotspots within hotspots. Elife.

[2] Bigoga J, Manga L, Titanji VPK, Coetzee M, Leke RGF (2007). Malaria vectors and transmission dynamics in coastal south-western Cameroon. Malar J.

[3] Ceccato P, Ghebremeskel T, Jaiteh M, Graves PM, Levy M, Ghebreselassie S (2007). Malaria stratification, climate, and epidemic early warning in Eritrea. Am J Trop Med Hyg.

[4] Chaves LF, Pascual M (2007). Comparing models for early warning systems of neglected tropical diseases. PLoS Negl Trop Dis.

[5] Cox J, Abeku TA (2007). Early warning systems for malaria in Africa: from blueprint to practice. Trends Parasitol.

[6] Githeko AK, Ogallo L, Lemnge M, Okia M, Ototo EN (2014). Development and validation of climate and ecosystem-based early malaria epidemic prediction models in East Africa. Malar J.

[7] Grover-Kopec E, Kawano M, Klaver RW, Blumenthal B, Ceccato P, Connor SJ (2005). An online operational rainfall-monitoring resource for epidemic malaria early warning systems in Africa. Malar J.

[8] Consortium Malaria (2007). Malaria: a hanbook for health professionals.

[9] Mubi M, Kakoko D, Ngasala B, Premji Z, Peterson S, Björkman A (2013). Malaria diagnosis and treatment practices following introduction of rapid diagnostic tests in Kibaha District, Coast Region, Tanzania. Malar J.

