# Supplementary material for: Increase in cases of malaria in Mozambique, 2014: epidemic or new endemic pattern?
Source: Rev Saude Publica. 2016 Mar 1;50:5. doi: 10.1590/S1518-8787.2016050006105 (PMC4794771; doi:10.1590/S1518-8787.2016050006105)
Supplement: Translation

# Translation: Aumento dos casos de malária em Moçambique, 2014: epidemia ou novo padrão de endemicidade?

**Authors:** Jorge Alexandre Harrison Arroz

**Affiliations:** World Vision Mozambique. Malaria Project Global Funded. Maputo, Moçambique

**Keywords:** Malária, epidemiologia, Doenças Endêmicas, Análise Espacial, Vigilância Epidemiológica, Estudos Transversais

## Abstract

**OBJECTIVO:**

Descrever o aumento de casos de malária em Moçambique.

**MÉTODOS:**

Estudo transversal conduzido em 2014, em Moçambique com dados do boletim epidemiológico semanal nacional. O número de casos registrados no período de 2009 a 2013 foi analisado e um canal endêmico foi criado usando o método de quartil e C-Sum. Taxas de incidência mensal foram calculadas para o primeiro semestre de 2014, permitindo determinar o padrão de endemicidade. Meses em que as taxas de incidência ultrapassaram o terceiro quartil ou a linha C-Sum foram declaradas como meses epidêmicos.

**RESULTADOS:**

As províncias de Nampula, Zambézia, Sofala e Inhambane foram responsáveis por 52,7% de todos os casos no primeiro semestre de 2014. Também durante esse período, as províncias de Nampula, Sofala e Tete foram responsáveis por 54,9% das mortes por malária. As taxas de incidência da malária em crianças, e todas as idades, registaram padrões na zona de epidemia. Para todas as idades, a taxa de incidência registou um pico em abril (2.573 casos/100 mil habitantes).

**CONCLUSÕES:**

Os resultados sugerem a ocorrência de um padrão epidêmico de malária no primeiro semestre de 2014 em Moçambique. É de importância estratégica haver vigilância mais acurada e em todos os níveis (distrital, provincial e central), para direccionar as intervenções de prevenção e controle em tempo útil.

## INTRODUÇÃO

A malária é endémica em Moçambique, representando 45,0% de todos os casos observados nas consultas externas e aproximadamente 56,0% de internamentos nas enfermarias de pediatria^a^. Segundo o último inquérito demográfico de saúde realizado em 2011 (IDS 2011), a prevalência da malária em crianças de seis a 59 meses é de 35,1%, sendo as províncias da Zambézia e Nampula aquelas com as mais elevadas prevalências (55,2% e 42,2%) e Maputo Cidade e Maputo Província, as mais baixas (2,5% e 4,8%) (Mapa 1)^b^.

Os principais vectores da malária em Moçambique pertencem aos grupos *Anopheles funestus* e *An. Gambiae*. O *Plasmodium falciparum* é o parasita mais frequente, responsável por cerca de 90,0% de todas as infecções maláricas, enquanto infecções por *Plasmodium malariae* e *Plasmodium ovale* são observadas em 9,0% e 1,0%, respectivamente^a^.

Vários factores contribuem para essa endemicidade, desde as condições climáticas e ambientais – como temperaturas e padrões de chuvas favoráveis – até locais propícios para a reprodução do vector, situação socioeconómica das populações relacionadas com a pobreza, habitações inapropriadas e acesso limitado aos meios de prevenção^a^.

Apesar das coberturas encorajadoras das principais intervenções de controlo, nomeadamente, distribuição de 2.252.192 redes mosquiteiras em campanhas, 89,0% de cobertura de redes para as gestantes e 79,0% de cobertura da pulverização intradomiciliar (PIDOM), o relatório do Programa Nacional de Controlo da Malária (PNCM) para o primeiro semestre de 2014 mostrou que foram notificados 3.297.386 casos e 1.937 óbitos em todo o País, aumento de 41,0% em relação aos casos notificados em igual período de 2013. O mesmo relatório refere uma ligeira redução dos óbitos por malária (2,0%) para o mesmo período de análise^c^.

O objetivo deste estudo foi descrever o aumento de casos da malária em Moçambique.

## MÉTODOS

Moçambique situa-se na zona austral e na costa oriental de África, tem uma superfície de aproximadamente 799.380 km^2^, faz fronteira a norte com a Tanzania, a ocidente com o Malawi, a Zambia, o Zimbabwe e a África de Sul, a sul com a Swazilandia e África de Sul e a este com oceano Índico^1^. Moçambique compreende uma larga faixa costeira com extensão aproximada de 2.515 km, banhada pelo Oceano Índico. Apresenta estação quente e húmida de outubro a março (caracterizada por chuvas, temperaturas e humidade relativa altas) e uma estação seca e fresca, de abril a setembro. No entanto, as condições climáticas variam de acordo com a altitude. Os valores diários de humidade relativa oscilam entre 10,0% a 90,0%. As temperaturas médias variam entre 20°C no sul e 26°C no norte, e os valores são mais elevados durante a época chuvosa^a^.

O País possui uma população estimada em 26.564.648 habitantes (em setembro de 2014)^d^ e está dividido em 10 províncias e uma capital provincial. As províncias da região norte do País são: Niassa, Cabo Delgado e Nampula; as províncias da região centro são: Zambézia, Tete, Manica e Sofala; as províncias da região sul são: Inhambane, Gaza, Maputo província e Maputo cidade (Figura 1).

**Figura 1:** Prevalência da malária por províncias. Moçambique, 2011.

Estudo descritivo transversal foi conduzido em setembro de 2014, com dados secundários provenientes do boletim epidemiológico semanal (BES). O BES é uma ferramenta do sistema de informação para a vigilância epidemiológica em Moçambique, direccionada para as principais doenças infecciosas que constituem problema de saúde pública no País. É usado em todas as unidades sanitárias do País, onde os dados produzidos são agregados ao nível distrital, provincial e nacional para serem analisados e estabelecida a tomada de decisão e acção em tempo útil.

Com recurso do BES nacional referente ao período 2009 e 2013, os dados foram agregados por meses e foi construído o canal endêmico para o período. Esse canal é um gráfico que permite estabelecer o padrão ou tendência de uma determinada doença ao longo do tempo, e dessa forma, detectar padrões de controlo, eliminação ou de epidemia da doença em causa. No presente estudo, foram adotadas duas metodologias para a construção do canal endêmico da malária para o período 2009 a 2013: método dos quartis e método da soma cumulativa (C-Sum)^e^.

O método de quartis é recomendado para definir o limiar de epidemia. Os dados referentes a casos de malária foram convertidos em taxas, usando a população do País como denominador de acordo com o ano em causa e expressas por 100.000 habitantes. Calculou-se o 1º, 2º e o 3º quartile, tendo gerado as seguintes zonas: zona de sucesso (abaixo do quartile 1); zona de segurança (entre quartile 1 e 2); zona de perigo/alerta (entre quartile 2 e 3); e zona epidêmica (acima do quartile 3).

O método C-Sum foi usado para controlo do método dos quartis. Para o cálculo do limiar para um determinado mês, somou-se o mês anterior e o posterior ao mês em causa, e o resultado foi dividido por 15. Por exemplo: para calcular o limiar de fevereiro, somou-se os dados de janeiro, fevereiro e março e dividiu-se o resultado por 15. O resultado é um gráfico com linha contínua (linha C-Sum) que determina o limiar da epidemia. Valores acima da linha C-Sum são considerados epidêmicos.

Taxas de incidências registadas mensais foram calculadas para o primeiro semestre de 2014, permitindo determinar o padrão da ocorrência da malária nesse período. Meses em que as taxas era superiores ao 3º quartile pelo método dos quartis e superiores à linha C-Sum foram considerados meses epidêmicos.

Usando os dados do PNCM referentes ao primeiro semestre de 2014, foi calculada a incidência registrada e mortalidade cumulativa por província, o que permitiu determinar as províncias mais afectadas pela malária.

## RESULTADOS

No primeiro semestre de 2014, as províncias de Nampula, Zambezia, Sofala e Inhambane foram responsáveis por 52,7% de todos os casos de malária em Moçambique e as províncias de Nampula, Sofala e Tete, responsáveis por 54,9% das mortes por malária. A taxa de letalidade variou entre 0,02% (Niassa) a 0,11% (Nampula e Cidade de Maputo) (Tabela).

**Tabela:** Casos, óbitos e taxa de letalidade por malária, segundo províncias. Moçambique, 1º semestre de 2014.

Província	Casos		Óbitos		Taxa de letalidade
		
	n*	%	n	%	%
Nampula	565.542	17,2	603	31,1	0,11
Zambézia	443.311	13,4	247	12,8	0,06
Sofala	365.121	11,1	213	11,0	0,06
Inhambane	364.498	11,1	180	9,3	0,05
Manica	363.340	11,0	173	8,9	0,05
Gaza	291.922	8,9	173	8,9	0,06
Cabo Delgado	253.351	7,7	123	6,4	0,05
Tete	243.332	7,4	80	4,1	0,03
Niassa	241.162	7,3	57	2,9	0,02
Província de Maputo	131.936	4,0	52	2,7	0,04
Cidade de Maputo	33.871	1,0	36	1,9	0,11

Moçambique	3.297.386	100	1.937	100	0,06

Fonte: PNCM (Programa Nacional de Controlo da Malária) – relatório do 1º semestre de 2014.

* Casos de infecção por *Plasmodium falciparum.*

A taxa de incidência registada da malária em menores de cinco anos mostra valores na zona epidêmica, passando de 5.739 casos por 100.000 habitantes em janeiro de 2014 a 4.508 casos por 100.000 habitantes em junho do mesmo ano, com um pico da taxa de incidência registada de 7.275 casos por 100.000 habitantes em abril (Figuras 2 e 3).

**Figura 2:** Canal endêmico 2009-2013 (crianças menores de 5 anos) pelo método dos quartis e taxas de incidência mensais registadas em 2014 (crianças menores de 5 anos), Moçambique.

**Figura 3:** Canal endêmico 2009-2013 (crianças menores de 5 anos) pelo método C-Sum e taxas de incidência mensais registadas em 2014 (crianças menores de 5 anos), Moçambique.

A taxa de incidência registada da malária para todas as idades mostra valores na zona epidêmica, que passaram de 2.020 casos por 100.000 habitantes em janeiro de 2014 a 1.482 casos por 100.000 habitantes em junho de 2014, com um pico da taxa de incidência registada de 2.573 casos por 100.000 habitantes em abril (Figuras 4 e 5).

**Figura 4:** Canal endêmico 2009-2013 (todas as idades) pelo método dos quartis e taxas de incidência mensais registadas em 2014 (todas as idades), Moçambique.

**Figura 5:** Canal endêmico 2009-2013 (todas as idades) pelo método C-Sum e taxas de incidência mensais registadas em 2014 (todas as idades), Moçambique.

## DISCUSSÃO

As províncias de Nampula e Zambézia foram as mais afectadas pela malária, o que pode estar relacionado com o facto de serem províncias costeiras com factores climáticos e socioeconómicos favoráveis (são as províncias mais populosas). Os achados deste estudo são consistentes com os dados nacionais, do IDS 2011, que revela as mais elevadas prevalências nacionais para essas províncias (Zambézia, 55,2% e Nampula, 42,2%)^b^. Vários estudos suportam uma maior dinâmica de transmissão da malária em regiões costeiras de África^1^ ^,^ ^2^ ^,^ ^8^ ^,^ ^9^. De acordo com os resultados, registou-se padrão epidêmico de malária desde janeiro de 2014, com pico em abril. O padrão epidêmico da malária é resultado de grandes alterações no sistema eco-epidemiológico, como chuvas excessivas, término de medidas de prevenção contra a malária, ou perda da eficácia das medidas adotadas por qualquer razão (resistência dos mosquitos aos insecticidas, falhas na implementação, quebras financeiras para sustentar as actividades de controlo da malária)^6^ ^,^ ^8^. Moçambique possui poucos dados disponíveis para a análise com acurácia da influência de factores climáticos. No entanto, a hipótese de uma maior visualização da porção do *iceberg* acima do horizonte clínico não pode ser descartada.

Desde 2006, o PNCM tem reforçado o sistema de notificação da malária por meio da melhoria do registo e da notificação de casos confirmados laboratorialmente ou pelo teste rápido de diagnóstico (TDRm)^f^. Em 2013, o PNCM disponibilizou 10.547.052 TDRm para testar cerca de 22 milhões de habitantes. Já em 2014 a quantidade foi maior (17.374.342), o que pode ter conduzido a uma maior capacidade de testagem, e assim, os resultados positivos serem registados e notificados (maior visualização da porção superior do *iceberg*)^f^.

Segundo o cartão de pontuação da eliminação da malária em África da ALMA (Aliança dos Líderes Africanos contra a Malária)^g^ ^,^ Moçambique deveria ter notificado sete milhões de casos em 2012 (o que corresponde a pelo menos três milhões e meio de casos num semestre), o que mostra que mesmo o presente aumento ainda representa uma subnotificação dos casos reais esperados. Analisando a curva epidêmica, pode-se observar que ela exibe padrão semelhante ao do canal endêmico, o que corrobora com a manutenção da mesma tendência sazonal, porém em faixa epidêmica. Isso sugere o aumento da capacidade de diagnóstico e registo pela maior disponibilidade de testes de diagnóstico. O aumento da rede sanitária não constitui motivo forte, pois expandiu em apenas 1,0% no primeiro semestre de 2014 em comparação com igual período de 2013, e corresponde a um aumento de 344 boletins epidemiológicos semanais recebidos (Relatório Anual do PNCM 2014)^e^.

De entre os vários propósitos de um sistema de vigilância epidemiológica, destacam-se: detectar epidemias; documentar a disseminação de doenças; prevenir e controlar a ocorrência de eventos adversos à saúde; e recomendar, com bases objectivas e científicas, as medidas necessárias para prevenir ou controlar a ocorrência de específicos agravos à saúde.

Não se pode entender como objetivo da vigilância a mera colecta e envio de dados^4^. O PNCM em Moçambique tem um sistema semanal de vigilância epidemiológica integrada ao BES, cobrindo todos os distritos e unidades sanitárias. No entanto, a análise dos dados de forma sistemática não é efectuada nos níveis distritais e provinciais. Outra limitação é que estes dados colhidos não reflectem a incidência real da malária no País, sendo apenas a incidência registada, o que pressupõe que muitos casos ainda não são diagnosticados e registados nos sistemas de informação em saúde.

Mesmo um canal endêmico para malária não está disponível a todos os níveis de atenção, facto que leva à detecção tardia de epidemias ou o risco de não as detectar. Vários países adoptam sistemas de vigilância combinados com dados de rotina dos hospitais e dados climáticos (precipitação, humidade relativa, temperatura) para a predição do aumento de casos com sucesso^3^ ^,^ ^5^ ^,^ ^7^.

Os resultados encontrados sugerem a ocorrência de um padrão epidêmico da malária durante o primeiro semestre de 2014 em Moçambique. A região norte e centro de Moçambique (Nampula, Zambézia e Sofala) foram as mais afectadas e que contribuíram significativamente para o aumento das taxas de incidência registada. As províncias de Inhambane e Gaza são as principais contribuintes para o aumento da taxa de incidência registada na região sul do País.

Os resultados também sugerem a importância estratégica de uma vigilância mais acurada e a todos os níveis para direccionar as intervenções de prevenção e controlo em tempo útil. Os distritos e províncias devem, no intuito de efectuar uma vigilância mais cerrada da malária, traçar os seus canais endêmicos e monitorar continuamente o reporte de casos de malária. Essa monitoria se estenderia ao nível central para dar maior suporte, em acções promotivas e preventivas, a áreas de risco de padrões epidêmicos e transformar a vigilância da malária numa intervenção principal associada ao acesso universal na prevenção, diagnóstico e tratamento de casos. Pesquisas básicas para impulsionar a inovação e o desenvolvimento de novas e melhores ferramentas de vigilância epidemiológica são necessárias e devem ser colocadas como tópico principal de uma agenda nacional de pesquisa em malária.

## Footnotes

a Ministério da Saúde (MZ), Direcção Nacional de Saúde Pública. Programa Nacional de Controlo da Malária: Plano Estratégico da Malária 2012-2016. Maputo; 2012 [citado 2015 set 10]. Disponível em: http://www.rollbackmalaria.org/files/files/countries/mozambique2012-2016.pdfb

b Instituto Nacional de Estatística (MZ); Ministério da Saúde (MZ). Inquérito demográfico e de saúde 2011. Maputo; 2013 [citado 2015 set 10]. Disponível em: http://dhsprogram.com/pubs/pdf/FR266/FR266.pdf

c Ministério da Saúde (MZ), Direcção Nacional de Saúde Pública. Programa Nacional de Controlo da Malária: relatório do 1º semestre de 2014*.* Maputo; 2014.

d Countrymeters. Contagem da População de Moçambique [citado 2014 set 15]. Disponível em: http://countrymeters.info/pt/Mozambique

e WHO. Epidemiological Approach for Malaria Control: Guide for tutors. 2nd.; 2013 [citado 2014 set 15]. Disponível em: http://apps.who.int/iris/bitstream/10665/96351/1/9789241506014_tutors_guide_eng.pdf

f Ministério da Saúde (MZ), Direcção Nacional de Saúde Pública. Programa Nacional de Controlo da Malária: relatório anual de 2014*.* Maputo; 2014.

g Aliança dos Líderes Africanos contra a Malária – ALMA. Cartão de Pontuação ALMA 2030 para a Eliminação da Malária [poster].Genebra: WHO; 2014 [citado 2015 set 10]. Disponível em: http://www.alma2015.org/sites/default/files/sadc-elimination-scorecard/alma_scorecards_poster_portuguese
